# Effect of Lower Extremity Bypass Surgery on Inflammatory Reaction and Endothelial Dysfunction in Type 2 Diabetic Patients

**DOI:** 10.1155/2009/417301

**Published:** 2009-04-05

**Authors:** Pei-Hsuan Tsai, Jun-Jen Liu, Szu-Yuan Chou, Yao-Chung Chang, Sung-Ling Yeh

**Affiliations:** ^1^School of Nutrition and Health Sciences, Taipei Medical University, Taipei 110, Taiwan; ^2^School of Medical Laboratory Science and Biotechnology, Taipei Medical University, Taipei 110, Taiwan; ^3^Department of Obstetrics and Gynecology, Taipei Medical University-Associated Wan Fang Hospital, Taipei 116, Taiwan; ^4^Department of General Surgery, Taipei Medical University-Wan Fang Hospital, Taipei 116, Taiwan

## Abstract

Diabetes mellitus (DM) is a metabolic disorder characterized by hyperglycemia and dyslipidemia. The abnormalities in nutrient metabolism and elevated inflammatory mediators resulting from DM lead to impairment of wound healing and vulnerability to infection and foot ulcers. Diabetic lower limb ischemia often leads to limb necrosis. Lower extremity bypass surgery (LEBS) is indicated to prevent limb loss in patients with critical leg ischemia. This study investigated the alteration of inflammatory and endothelium dysfunction markers before and after LEBS in DM patients. Twenty one type 2 DM patients with LEBS were included. Blood was drawn before and at 1 day and 7 days after surgery in the patients. Plasma soluble cellular adhesion molecule levels and blood leukocyte integrin expressions were measured. Also, plasma concentrations of endothelin-1 and nitric oxide were analyzed to evaluate the vascular endothelial function. The results showed that there were no significant differences in plasma cellular adhesion molecules, endothelin-1 and nitric oxide levels, nor did any differences in leukocyte integrin expressions before and after the operation. These results suggest that the efficacy of LEBS on alleviating inflammatory reaction and improving endothelial function in DM patients was not obvious.

## 1. Introduction

Diabetes mellitus (DM) was the 4th leading cause of death in Taiwan in 2007. Many diabetic patients have an increased risk of atherosclerosis, cerebrovascular disease, and peripheral vascular diseases [1]. The abnormalities in nutrient metabolism and elevated inflammatory mediators resulting from DM lead to impairment of wound healing and vulnerability to infection and foot ulcers. Diabetic lower limb ischemia caused by arterial occlusion is the most common foot injury leads to lower extremity amputation in DM patients [2]. Lower extremity bypass surgery (LEBS) is indicated to prevent limb loss in patients with critical leg ischemia. Previous studies revealed that a large reduction in major amputation rates is associated with the increase of LEBS [3, 4]. However, the efficacy of LEBS on inflammatory reaction in DM patients has not been evaluated and the changes in inflammatory mediators before and after the LEBS remain unknown.

Endothelial dysfunction accompanied by upregulated inflammatory mediators is a major contributing factor to the pathogenesis of diabetic vascular complications [5]. The injured vasculature endothelium promotes the expressions of cellular adhesion molecules. Overexpressions of cellular adhesion molecule facilitate leukocyte-endothelial interactions which may aggravate inflammatory reaction and tissue damage [6]. Previous study demonstrated that increased levels of plasma soluble adhesion molecule occur in type 2 patients [7]. Endothelin-1 (ET-1) is a potent vasoconstrictor with mitogenic property. ET-1 stimulates vascular smooth muscle cells proliferation, a major step in the development of atherosclerosis [8]. Nitric oxide (NO) is a vasodilator produced by endothelial cells. Other key roles of NO include inhibiting platelet aggregation, smoothing muscle cells proliferation, reducing monocyte adherence, and so forth [9]. Previous study showed that at the onset of diabetes, the release or response to NO is reduced [10]. Both ET-1 and NO are important mediators in maintaining vascular functions. Since LEBS increases blood flow to tissues that may carry oxygen and nutrients to the lower extremities and improve the healing of the tissues [11], we hypothesized that the inflammatory process and vascular dysfunction are attenuated in DM patients undergoing LEBS. Therefore, the aim of this study was to investigate whether reperfusion of the lower extremities may improve the inflammation and endothelial dysfunction in DM patients.

## 2. Subjects and Methods

### 2.1. Subjects

This study was conducted from April to December 2006 at Taipei Medical University-affiliated Wan Fang Hospital. Twenty one type 2 DM patients with severe diabetic lower limb ischemia and underwent LEBS were enrolled in the experimental group. The diabetes duration was 6–30 years, with a mean of 18.2 years. Insulin and combined therapy if necessary were used to control blood glucose to within a range of 106–259 mg/dL. No leg infection was observed in LEBS patients, possibly because the operation was successful and the antibiotics used after the surgery. The protocol was approved by the hospital ethics committee, and all the patients gave their informed written consent prior to their participation in this study.

### 2.2. Blood Sampling

Blood samples were taken from each patient before and at 1 day and 7 days after the LEBS in DM patients. Ten milliliters of blood were drawn after 12 hours of fasting, placed in tubes containing ethylene diaminetetraacetic acid. Fresh blood samples were collected for the analysis of leukocyte CD11a/CD18 and CD11b/CD18 expressions. Plasma samples obtained from whole blood centrifugation at 3000 rpm for 10 minutes were stored at −80°C until further analysis.

### 2.3. Measurements and Analytical Procedures

Total cholesterol, low-density lipoprotein cholesterol, high-density lipoprotein cholesterol, triglyceride, and creatinine were analyzed by an autoanalyser (Hitachi 7170, Tokyo, Japan). Blood HbA1C was measured using a commercial kit (Helena BioSciences, sunderland, UK). Procedures followed the manufacturer instructions.

### 2.4. Measurements of Plasma ET-1, NO, sICAM-1, sVCAM-1, and C-reactive Protein Concentrations

Concentrations of plasma ET-1, soluble intracellular adhesion molecule (sICAM)-1, soluble vascular cell adhesion molecule (sVCAM)-1, and C-reactive protein (CRP) were measured using commercial enzyme-linked immunosorbent assay (ELISA) kits (R&D systems, Minneapolis, MN, USA). Concentrations of NO_2_^−^/NO_3_^−^ were determined with a commercial kit (R&D systems). Procedures followed the manufacturer instructions. The minimum detectable dose of ET-1, sICAM-1, sVCAM-1, and CRP were 0.064 pg/mL, 0.35 ng/mL, 0.6 ng/mL, 1 ug/ml, respectively, for NO_2_^−^/NO_3_^−^ was 0.25 uM.

### 2.5. Analysis of Lymphocyte CD11a/CD18 and Polymorphoneuclear Neutrophil CD11b/CD18 Expressions

One hundred microliters of fresh blood was incubated with 10 uL fluorescent isothiocynate (FITC)-conjugated mouse monoclonal antihuman CD11a and phycoerthrin (PE)-conjugated mouse monoclonal antihuman CD18 (Serotec, Oxford, UK) for 15 minutes at 4°C. The proportions of CD11a/CD18 expressed on lymphocytes were analyzed by flow cytometry (Coulter, Miami, FL). The results are presented as a percentage of CD11a-presenting cells in 1 × 10^5^ lymphocytes. To measure CD11b/CD18 expressions on polymorphoneuclear neutrophils (PMNs), FITC-conjugated mouse monoclonal antihuman CD11b and PE-conjugated mouse monoclonal antihuman CD18 (Serotec) were added into 100 *μ*L of fresh blood. The results are presented as a percentage of CD11b/CD18 expression in 1 × 10^5^ PMNs. Lymphocytes and PMNs were gated on the basis of the forward scatter and side scatter profiles and were analyzed for the expressions of CD11a/CD18 and CD11b/CD18, respectively.

### 2.6. Statistical Analysis

Data are presented as mean ± SD. All statistical analyses were performed using SAS software package. The differences among different time points were determined by one-way analysis of variance. *P* < .05 was considered statistically significant.

## 3. Results

The characteristics of the subjects were presented in Table 1. There were no significant differences in plasma ET-1 and NO levels (Table 2), nor did any differences in plasma sVCAM, sICAM, and CRP levels before and after the operation (Table 3). Also, leukocyte CD11a/CD18 and CD11b/CD18 expressions before and after the operation did not differ in DM patients (Table 4).

## 4. Discussion

This study evaluated an LEBS-induced change in inflammatory response and endothelial function. We found that compared with the preoperative condition, the mediators related to inflammation and vascular function did not change in type 2 DM patients at early and late stages after LEBS.

Diabetic patients often have endothelial dysfunction and releasing of endothelins is partly responsible for this. Previous report found that plasma ET-1 levels are enhanced in patients with poor glycemic control [12] and ET-1 levels were even higher in DM patients complicated with vascular diseases [13]. A study performed by Schneider et al. [14] found that diabetic patients taking angiotensin converting enzyme inhibitors had lower plasma ET-1 levels than patients without, indicating that medical intervention did improve ET-1 levels. In addition to ET-1, NO is also an important regulatory determinant of vascular tissue homeostasis. NO plays a protective role by suppressing abnormal proliferation of vascular smooth muscle following various vascular interventions such as bypass grafting [15]. In this study, we did not observe differences in plasma ET-1 and NO levels before and 1 or 7 days after surgery. This result may indicate that LEBS performed in this study did not, at least in the short run, improve endothelial function in DM patients.

CRP is an inflammatory marker. CRP levels were correlated with peripheral artery disease severity in patients undergoing LEBS [16]. ICAM-1 and VCAM-1 are adhesion proteins synthesized by endothelial cells. Their expressions greatly increase after stimulation by proinflammatory cytokines [17]. Previous study showed that tissue ICAM-1 levels were positively correlated with blood glucose levels [18]. Adhesive interactions between leukocytes and endothelial cells are involved in inflammatory or immunologic response mechanisms. Adhesion molecules on endothelial cells are the ligands of integrins on leukocytes. CD11a and CD11b/CD18 are members of the leukocyte adhesion molecules *β*_2_ integrin. CD11a/CD18 are exclusively expressed on leukocytes and CD11b/CD18 are abundant in PMNs [19]. In this study, we analyzed lymphocyte CD11a/CD18 because the function of T-lymphocyte subsets is important on influencing the type of immunity and the inflammatory response to infection [20]. CD11b/CD18 expressed by neutrophil is important in mediating neutrophil-endothelial cell interactions and binding to adhesion molecule on the surface of vascular endothelial cells [21, 22]. Previous reports revealed that neutrophil CD11b/CD18 increases in infected patients and is correlated with microvascular dysfunction [23]. In this study, we did not observe the difference in leukocyte CD11a/CD18 and CD11b/CD18 expressions before and after the operation. This result was consistent with plasma sVCAM-1, sICAM-1, and CRP levels that these inflammatory proteins did not change after LEBS. These findings indicate that compared with preoperative state, LEBS did not attenuate inflammatory reaction in DM patients. Surgery and trauma induce a generalized state of inflammation. Although LEBS increases blood flow to the peripheral tissues, surgical injury stimulates the production of endogenous inflammatory mediators. Besides, reperfusion of the ischemic tissues may also result in exaggerated inflammatory response [24, 25], this may make the alteration of inflammatory mediators not so obvious before and after the surgery.

In conclusion, this is a pilot study to demonstrate that compared with the preoperative condition, no differences in plasma concentrations of ET-1, NO, and inflammatory mediators were observed in DM patients after LEBS. This result suggests that the effect of LEBS on alleviating the inflammatory reaction and improving endothelial function in DM patients was not obvious.

## Figures and Tables

**Table 1:** Characteristics of the subjects.

	Diabetic patients
Age (yr)	70.7 ± 9.0
Gender (M/F)	10/11
Blood glucose (mg/dL)	175.9 ± 59.5
HbA1C (%)	8.7 ± 2.3
Total cholesterol (mg/dL)	152.9 ± 35.6
HDL-C (mg/dL)	32.7 ± 13.1
LDL-C (mg/dL)	86.0 ± 33.2
Triglyceride (mg/dL)	145.8 ± 81.6
Creatinine (mg/dL)	3.70 ± 3.44

Data are expressed as the mean ± SD. Abbreviations: HbA1C: Hemoglobin A1C; HDL-C: High-density lipoprotein-cholesterol; LDL-C: Low-density lipoprotein-cholesterol.

**Table 2:** Plasma endothelin (ET)-1 and nitric oxide (NO) concentrations before and after the surgery.

Days	ET-1	NO
	(pg/mL)	(umol/L)
0	2.2 ± 0.5	3.5 ± 1.7
1	2.0 ± 0.2	2.5 ± 0.7
7	2.2 ± 0.4	3.4 ± 1.9

Data are expressed as the mean ± SD.

**Table 3:** Plasma soluble intercellular adhesion molecule (sICAM), soluble vascular cell adhesion molecule (sVCAM), and C-reactive protein (CRP) concentrations before and after the surgery.

Days	sVCAM	sICAM	CRP
	(ng/mL)		(ug/mL)
0	1406.2 ± 839.7	344.3 ± 121.8	53.4 ± 46.7
1	1415.9 ± 722.6	332.3 ± 112.1	64.6 ± 54.9
7	1224.9 ± 632.8	398.5 ± 126.9	48.3 ± 45.7

Data are expressed as the mean ± SD.

**Table 4:** Expressions of lymphocyte CD11a/CD18 and neutrophil CD11b/CD18 before and after the surgery.

Days	CD11a/CD18	CD11b/CD18
	(%)	
0	48.2 ± 18.0	3.1 ± 2.0
1	42.4 ± 12.0	2.9 ± 1.3
7	43.5 ± 12.3	3.5 ± 1.1

Data are expressed as the mean ± SD.
